# Mediterranean Diet and Breast Cancer Risk

**DOI:** 10.3390/nu10030326

**Published:** 2018-03-08

**Authors:** Federica Turati, Greta Carioli, Francesca Bravi, Monica Ferraroni, Diego Serraino, Maurizio Montella, Attilio Giacosa, Federica Toffolutti, Eva Negri, Fabio Levi, Carlo La Vecchia

**Affiliations:** 1 Department of Clinical Sciences and Community Health, Università degli Studi di Milano, 20133 Milan, Italy; federica.turati@unimi.it (F.T.); greta.carioli@unimi.it (G.C.); francesca.bravi@unimi.it (F.B.); monica.ferraroni@unimi.it (M.F.); 2 Unit of Cancer Epidemiology, CRO Aviano National Cancer Institute, IRCCS, 33080 Aviano, Italy; serrainod@cro.it (D.S.); toffolutti@gmail.com (F.T.); 3 Epidemiology and Biostatistical Unit, Istituto Tumori “Fondazione Pascale IRCCS”, 80131 Naples, Italy; m.montella@istitutotumori.na.it; 4 Department of Gastroenterology and Clinical Nutrition, Policlinico di Monza, 20900 Monza, Italy; attilio.giacosa@policlinicodimonza.it; 5 Department of Biomedical and Clinical Sciences, Università degli Studi di Milano, 20157 Milan, Italy; eva.negri@unimi.it; 6 Institute of Social and Preventive Medicine (IUMSP), Lausanne University Hospital, 1010 Lausanne, Switzerland; fabio.levi@chuv.ch

**Keywords:** Mediterranean diet, breast cancer, prevention, case-control

## Abstract

The Mediterranean diet has been related to a reduced risk of several common cancers but its role on breast cancer has not been quantified yet. We investigated the association between adherence to the Mediterranean diet and breast cancer risk by means of a hospital-based case-control study conducted in Italy and Switzerland. 3034 breast cancer cases and 3392 controls admitted to the same network of hospitals for acute, non-neoplastic and non-gynaecologic diseases were studied. Adherence to the Mediterranean diet was quantitatively measured through a Mediterranean Diet Score (MDS), summarizing the major characteristics of the Mediterranean dietary pattern and ranging from 0 (lowest adherence) to 9 (highest adherence). We estimated the odds ratios (ORs) of breast cancer for the MDS using multiple logistic regression models, adjusting for several covariates. Compared to a MDS of 0–3, the ORs for breast cancer were 0.86 (95% confidence interval, CI, 0.76–0.98) for a MDS of 4–5 and 0.82 (95% CI, 0.71–0.95) for a MDS of 6–9 (*p* for trend = 0.008). The exclusion of the ethanol component from the MDS did not materially modify the ORs (e.g., OR = 0.81, 95% CI, 0.70–0.95, for MDS ≥ 6). Results were similar in pre- and post-menopausal women. Adherence to the Mediterranean diet was associated with a reduced breast cancer risk.

## 1. Introduction

Despite considerable research, the role of diet in the aetiology of breast cancer is still an open issue. Apart from a consistent direct association with alcohol intake [1,2], evidence on other specific foods or nutrients is controversial, with weak and inconsistent associations often reported [3,4]. Nevertheless, investigating the relation of dietary patterns with breast cancer remains of great interest, taken into account that cancer prevention through dietary and lifestyle choices has been identified as a priority for cancer research [5].

Dietary patterns assess diet as a whole and capture the complex inter-relationships among dietary components and their cumulative effects. Therefore, they may be more predictive of disease risk than individual foods or nutrients. However, the available evidence based on dietary patterns is still limited [6]. The Mediterranean dietary pattern, characterized by an abundance of plant foods, the use of olive oil as the principal source of fat and moderate consumption of meat, fish and wine [7,8], has been favourably related to all-cause mortality and several chronic diseases [9], including coronary heart diseases [9], cerebrovascular diseases [10], diabetes [11], cancer overall [12] and selected common cancers [13], mainly those of the digestive tract [14,15,16].

An inverse association between this dietary pattern and breast cancer has been suggested by some studies [17,18,19] but others failed to confirm such observation [20,21,22], or reported inverse associations confined to specific cancer subgroups defined by hormone receptor status [23,24,25] or menopausal status [23,26,27]. A meta-analysis updated in August 2017 and including studies using a priori developed Mediterranean diet scores, as well as a posteriori-defined Mediterranean dietary patterns indicated an inverse association overall (pooled relative risk, RR, for the study-specific highest versus lowest category = 0.92, 95% confidence interval, CI, 0.89–0.96) and among postmenopausal women (pooled RR = 0.92, 95% CI, 0.86–0.99), but not premenopausal ones (pooled RR = 1.3) [28]. In addition, another meta-analysis with a comparable update, focusing on postmenopausal breast cancer only and considering prospective studies only, provided a similar summary point estimate (RR = 0.94), which, however, was only marginally significant (95% CI, 0.88–1.01) [24].

A protective role of the Mediterranean diet on breast cancer is biologically plausible since the Mediterranean dietary pattern is rich in fibre, antioxidants, including flavonoids, vitamins, carotenoids and squalene (mainly from virgin olive oil). It may modulate breast cancer risk by decreasing endogenous oestrogens [29], increasing sex-hormone binding globulin levels [18], neutralizing free radicals and preventing DNA damage [30,31], and reducing oxidative stress [32,33].

In addition, promising results suggesting a beneficial role of the Mediterranean diet in the prevention of (postmenopausal) breast cancer came from the PREDIMED randomized trial, which estimated a hazard ratio of 0.32 (95% CI, 0.13–0.79) for the Mediterranean diet with extra-virgin olive oil group and of 0.59 (95% CI, 0.26–1.35) for the Mediterranean diet with nuts group, versus the control diet group [34].

Given the inconsistent results and the fact that several studies on the topic were conducted outside the Mediterranean region, where a high compliance with the Mediterranean diet is unlikely, it is worthwhile to provide new epidemiological data on the association between this dietary profile and breast cancer in a Mediterranean setting. We have therefore evaluated if a high adherence to the Mediterranean diet is associated with a decreased risk of breast cancer in a large study undertaken in Italy and French-speaking Switzerland, where large segments of the population still adhere to the Mediterranean diet.

## 2. Materials and Methods

Study population and data collection. Data were from a multicentric case-control study on breast cancer conducted in 6 Italian areas (i.e., Milan, Genoa, Pordenone/Gorizia, Forlì, Latina and Naples) in 1991–2008 and in the Canton of Vaud, Switzerland, in 1992–2008 [35,36]. Overall, cases were 3034 women (median age 55, range: 23–78 years) with incident, histologically confirmed breast cancer diagnosed in the year before the interview and without history of cancer at other sites, admitted to major teaching and general hospitals in the study areas. Controls were 3392 patients (median age 56, range: 19–79 years) admitted to the same hospitals for a wide spectrum of acute, non-neoplastic, non-gynaecological conditions and with no previous cancer or recent changes of the diet. Among controls, 30% was admitted for acute surgical conditions, 20% for traumas, 26% for non-traumatic orthopaedic conditions, 14% for eye disorders and 10% for other miscellaneous conditions. According to the local ethical committees’ requirements at the time of data collection, informed consent was obtained from each patient. Less than 5% of cases and controls identified in Italy and about 15% of those from Switzerland refused to be interviewed.

Centrally trained interviewers administered to cases and controls a structured questionnaire, including information on sociodemographic characteristics and anthropometric measurements (e.g., self-reported weight and height), lifestyle habits (e.g., tobacco smoking, alcohol drinking and physical activity), personal medical history, menstrual and reproductive factors, oral contraceptive use, menopausal hormone-replacement therapy use and family history of cancer. Information on dietary habits before diagnosis (for cases) or hospital admission (for controls) was based on a validated and reproducible food frequency questionnaire (FFQ) [37,38,39] including information on 78 foods and beverages, as well as a range of the most common recipes. For each dietary item, participants were asked to report their average weekly frequency of consumption. A specific section collected information of weekly consumption of different types of alcoholic beverages. Frequencies reported less than once per week but at least once per month, i.e., occasional intakes, were coded as 0.5 per week. Sporadic consumptions were not considered. An Italian food composition database was used to estimate nutrient and total energy intake [40,41].

Mediterranean Diet Score (MDS). The adherence to the Mediterranean diet was assessed through an a priori score (Mediterranean diet score, MDS), developed by Trichopoulou and colleagues [42], which included nine dietary components: fruit, vegetables, cereals (including bread and potatoes), legumes, fish, monounsaturated/saturated fatty acid ratio, dairy products, meat (including meat products) and alcoholic beverages. For each study subject and for each score component, a value of 0 or 1 was attributed as follows: for components frequently consumed in the traditional Mediterranean diet (i.e., fruit, vegetables, cereals, legumes, fish and high monounsaturated/saturated fatty acid ratio), subjects were assigned a value of 1 if they had a consumption above or equal to the study-specific median among controls and 0 otherwise; for components less frequently consumed in the Mediterranean diet (i.e., dairy and meat products), participants with a consumption below the study-specific median were assigned a value of 1 and 0 otherwise. For alcohol, 1 point was attributed to women consuming 5 g to less than 25 g of ethanol per day and 0 otherwise. We then calculated the MDS adding up the points for each of the nine individual binary components; thus, the score varied between 0 and 9, the higher the score the stronger the adherence to Mediterranean diet.

Statistical analysis. We estimated odds ratios (ORs) of breast cancer and their corresponding 95% CIs for categories of MDS (0–3, 4–5, 6–9) and for an increment of one point in the MDS using unconditional multiple logistic regression models. We included terms for study centre, age (quinquennial; categorically), education (<7, 7–11, ≥12 years; categorically), body mass index (BMI, <20, 20–24.9, 25–29.9, ≥30 kg/m^2^; categorically), level of physical activity at workplace/home at age 30–39 (at age 19–30 for the few women aged < 30) (low, medium, high; categorically) [43,44], tobacco smoking (never smoker, former smoker, current smoker of <15 and current smoker of ≥15 cigarettes/day; categorically), parity (0, 1, 2, 3, ≥4; categorically), menopausal status and age at menopause (pre-/peri-menopause, menopause at <50 and menopause at ≥50 years of age; categorically), oral contraceptive use (ever, never), hormone-replacement therapy use (ever, never), history of diabetes, family history of breast cancer in first degree relatives, and non-alcohol energy intake (study-specific quintiles according to control distribution; categorically). Test for trend was performed by including the MDS as ordinal variable. Since alcohol is a recognized risk factor for breast cancer [1], in a further analysis we recalculated the MDS without the alcohol component and included alcohol as a confounder in the logistic regression model.

We conducted analyses stratified by age, menopausal status, education, parity, BMI, oral contraceptive use and hormone-replacement therapy use. Heterogeneity across strata was tested by likelihood ratio tests.

We computed the population attributable fraction (PAF) to estimate the proportion of breast cancer cases that might have been avoided by shifting all subjects to the highest MDS category (i.e., 6–9 points), according to the method of Bruzzi et al. [45]. In addition, we estimated preventable proportions associated with shifting all subjects to the adjacent lower risk category (i.e., adjacent upper category of the MDS).

All the analyses were performed using the SAS software, version 9.4 (SAS Institute, Inc., Cary, NC, USA).

## 3. Results

Table 1 shows the distribution of 3034 breast cancer cases and 3392 controls according to selected covariates. Compared to controls, cases were more educated, more frequently in pre-menopause, had a lower parity and reported more frequently a family history of breast cancer. Supplementary Materials Table S1 describes the characteristics of cases and controls, separately for Italy and Switzerland. Swiss women were more educated and tended to be more nulliparous as compared to Italian women. No major differences emerged for age, menopausal status and family history of breast cancer.

Among controls, the median values used to define adherence for single components were 17.5 weekly portions for fruit, 11.0 for vegetables, 22.5 for cereals, 0.5 for legumes, 1.5 for fish, 1.3 for monounsaturated/saturated fatty acid ratio, 10.1 for dairy products and 5.8 for meat. No major differences emerged in the distribution of these dietary items between Italian and French speaking Swiss women.

Table 2 illustrates the adjusted ORs of breast cancer according to the MDS. Compared to the lowest (0–3) category, the ORs were 0.86 (95% CI 0.76–0.98) for MDS of 4–5 and 0.82 (95% CI 0.71–0.95) for MDS of 6–9, with a significant trend of decreasing risk (*p*-value = 0.008). The OR for one point increment in the MDS was 0.95 (95% CI 0.92–0.99). When we excluded the alcohol component from the score, the ORs were 0.81 (95% CI, 0.71–0.91) for 4–5 and 0.81 (95% CI, 0.70–0.95) for 6–8 components. Estimation of the PAF indicated that 7.2% of breast cancer cases in this population would be avoided by shifting all women towards the highest category of the score (i.e., 6–9). The PAF associated to the one category upward shift of subjects in the lowest and middle categories of MDS (i.e., 0–3 and 4–5, respectively) was 6.1%.

The association between the MDS and breast cancer risk in subgroups of potential effect modifiers is described in Table 3. In general, ORs estimates were not significantly different across strata. The inverse associations appeared somewhat stronger among more educated, overweight women and women using HRT, but the heterogeneity tests were significant for HRT only.

## 4. Discussion

By using a simple, intuitive and frequently used dietary score to assess compliance to the Mediterranean diet, this large investigation from southern Europe found that adherence to the Mediterranean dietary pattern is related to a reduced risk of breast cancer, as documented for several other cancers [13]. In particular, women in the highest category of adherence of the MDS (i.e., 6–9 points) had an approximately 20% decreased risk compared to women in the lowest one (i.e., 0–3 points). Moreover, we estimated that 6% of breast cancer cases in this population would be avoided by shifting all women to the upper adjacent category of the MDS. In stratified analyses, including those by menopausal status, the results were similar and many of the variations in the ORs may have been due to chance.

Available epidemiological data on Mediterranean diet and breast cancer appear conflicting, with some studies reporting null findings [20,21,22], some inverse associations [17,18,19], generally of weak magnitude, and others inverse associations restricted to specific subgroups, in particular postmenopausal women [23,27] and oestrogen receptor—(ER) negative breast cancer cases [23,24,25].

Among major prospective investigations, the Nurses’ Health Study (with 3580 breast cancer cases) did not find a relation between Mediterranean diet and total or ER-positive breast cancer but an inverse association was observed for ER-negative breast cancer [25]. The HRs for the upper quintile of a modified version (aMED) of the Mediterranean diet scale by Trichopoulou et al. [42] were 0.98 (95% CI, 0.88–1.10) for total, 1.05 (95% CI, 0.91–1.18) for ER-positive and 0.79 (95% CI, 0.60–1.03, *p* for trend = 0.03) for ER-negative breast cancer [25]. Similar findings were reported from postmenopausal women participating in the Netherlands Cohort study: based on 2321 incident breast cancer cases, a significant inverse association with a Mediterranean diet score without alcohol was observed for ER-negative breast cancer (HR for 6–8 vs. 0–3 points: 0.60, 95% CI, 0.39–0.93, *p* trend = 0.032), whereas the associations with total (HR: 0.87, 95% CI, 0.72–1.06) and ER-positive breast cancer (HR: 0.87, 95% CI, 0.69–1.10) were weak and non-significant [24]. In addition, based on approximately 9000 postmenopausal and 1200 premenopausal women with incident breast cancer from 10 European countries, the European Prospective Investigation into Cancer and nutrition cohort (EPIC) found an inverse association between a 16-point Mediterranean diet score which excluded the alcohol component and the risk of overall breast cancer (HR for the highest vs. lowest category: 0.94, 95% CI, 0.88–1.00, *p* trend = 0.048), which appeared limited to postmenopausal breast cancer (HR: 0.93, 95% CI, 0.87–0.99, *p* trend = 0.037), compared to premenopausal breast cancer (HR: 0.97, 95% CI, 0.81–1.15) [23]. Conversely, in a British cohort of almost 34,000 women and 828 incident breast cancer cases, a non-significant inverse association with increasing adherence to the Mediterranean diet emerged only among premenopausal women (HR = 0.65, 95% CI, 0.42–1.02 for the highest versus lowest Mediterranean score category, *p* for trend = 0.09) [26]. Findings from the Swedish Women’s Lifestyle and Health cohort, based on 1278 incident cases, indicated an increased breast cancer risk for a higher Mediterranean diet score, but results became not significant when alcohol was excluded from the score [21]. When findings from the 5 cohort studies with data on postmenopausal breast cancer published up to August 2016 were pooled in a meta-analysis, a marginally significant 6% reduced risk of postmenopausal breast cancer was estimated for the highest versus the lowest category of scores of the Mediterranean diet (RR = 0.94, 95% CI, 0.88–1.01) [24].

Among case-control studies, an investigation among Asian American women with 1248 breast cancer cases found a significant inverse association with a score of Mediterranean diet which considered alcohol among the non-Mediterranean components (OR for the upper vs. the lower score categories: 0.65, 95% CI, 0.44–0.95, *p* for trend = 0.009) [18]; a Spanish case-control study with about 1000 cases found a non-significantly reduced breast cancer risk for higher scores in the aMED excluding alcohol (OR: 0.74, 95% CI, 0.46–1.18, *p* trend = 0.01) and a significant 11% decreased risk per one standard deviation increase in the score [19]; and a study on approximately 1000 Greek-Cypriot postmenopausal women found no association with two different a priori Mediterranean diet scores, modified in their scoring system as to giving higher scores to no alcohol consumption [22].

Thus, there is a partial consensus of findings on Mediterranean diet and breast cancer between cohort and case-control studies. This strengthens the consistence of a real inverse association. Available studies were conducted on different populations and they measured adherence to the Mediterranean diet through various scores, which differed in the components included (e.g., dairy foods, fish), the specific food items considered in a component (e.g., only whole grain products in the cereal component, only red meat in the meat component) and the scoring system for the level of adherence (0/1, or finer scoring systems). Moreover, while moderate alcohol intake is a distinctive feature of the Mediterranean diet, given its unfavourable role on breast cancer risk, some studies excluded alcohol from the score calculation [19,23] and some considered alcohol among the negative components [18,20,22]. In our study, the exclusion of ethanol intake from the score did not materially modify any of the ORs. In addition, differences in eating and lifestyle habits between Mediterranean and non-Mediterranean populations can influence the evaluation of the health benefits of this specific dietary pattern [46]. Indeed, Mediterranean diet scores may be difficult to interpret when applied to non-Mediterranean populations if there are relevant differences between Mediterranean and non-Mediterranean countries on the absolute amounts of food intakes. The cut-offs used to define adherence are population-dependent in several scores and may not be able to discriminate beneficial/harmful levels of intakes in non-Mediterranean countries. The varieties of foods/food pattern within a single dietary component (e.g., different varieties of vegetables/fruits, olive oil versus consumption of animal products as the major source of monounsaturated fatty acid, (red) wine during meals versus binge drinking mostly of beer/spirits), may also differ. Thus, the application of a Mediterranean dietary score to a population with a very different dietary pattern may not capture a real Mediterranean dietary style [47].

Strengths of our study include the large sample size, the Mediterranean setting, the comparable catchment areas of cases and controls, the very high participation, the reproducibility [37,39] and validity [38] of information on diet and the allowance for a large number of covariates. Although Switzerland is not considered a typical Mediterranean area, the distribution of the dietary components included in the MDS score among Swiss women was similar to that of the Italian population. Comparison across countries revealed that Swiss women were more educated and tended to be more nulliparous compared to Italian ones, while no major differences emerged for age, menopausal status and family history of breast cancer.

Among the limitations, we had no adequate information on various subtypes of breast cancer. Selection bias should be limited, as we excluded from the control group subjects admitted to hospitals for chronic and gynaecologic conditions or diseases related to diet modifications or known risk factors for breast cancer. Information bias was minimized by the direct interview of cases and controls by the same trained interviewers in similar hospital conditions.

## 5. Conclusions

In conclusion, we found a modest but relevant at both individual and public health level, reduced risk of breast cancer for a high adherence to the Mediterranean diet, in Southern European populations where this dietary pattern is still prevalent.

## Figures and Tables

**Table 1:** Distribution of 3034 cases of breast cancer and 3392 controls according to selected characteristics. Italy and Switzerland, 1991–2008.

Characteristic	Cases *n* (%)	Controls *n* (%)
Centre		
Pordenone/Gorizia	1046 (34.5)	1015 (29.9)
Milan	585 (19.3)	623 (18.4)
Genoa	290 (9.6)	310 (9.1)
Forlì	212 (7.0)	213 (6.3)
Naples	258 (8.5)	249 (7.3)
Rome/Latina	178 (5.9)	178 (5.3)
Switzerland	465 (15.3)	804 (23.7)
Age group		
<40	260 (8.6)	403 (11.9)
40–44	302 (10.0)	283 (8.3)
45–49	416 (13.7)	381 (11.2)
50–54	482 (15.9)	489 (14.4)
55–59	467 (15.4)	498 (14.7)
60–64	445 (14.7)	480 (14.2)
65–69	413 (13.6)	483 (14.2)
≥70	249 (8.2)	375 (11.1)
Education (years) ^a^		
<7	1273 (42.2)	1583 (47.0)
7–11	972 (32.2)	1120 (33.2)
≥12	775 (25.7)	666 (19.8)
Menopausal status ^a^		
Pre-/peri- menopause	1150 (38.0)	1180 (34.8)
Menopause at <50 years of age	777 (25.7)	1018 (30.0)
Menopause at ≥50 years of age	1097 (36.3)	1191 (35.1)
Parity ^a^		
Nulliparae	504 (16.6)	597 (17.6)
1	676 (22.3)	688 (20.3)
2	1163 (38.4)	1179 (34.8)
3	465 (25.3)	583 (17.2)
≥4	223 (7.4)	343 (10.1)
Family history of breast cancer in first-degree relatives		
No	2724 (89.8)	3249 (95.8)
Yes	310 (10.2)	143 (4.2)

^a^ The sum does not add up to the total because of missing values.

**Table 2:** Distribution of 3034 breast cancer cases and 3392 controls, odds ratios (OR) and 95% confidence intervals (CI) for the Mediterranean diet score (MDS). Italy and Switzerland, 1991–2008.

	Cases	(%)	Controls	(%)	OR (95% CI) ^a^
**MDS ^b^**					
0–3	877	(28.9)	1022	(30.2)	1 ^c^
4–5	1323	(43.6)	1482	(43.7)	0.86 (0.76–0.98)
6–9	833	(27.5)	886	(26.1)	0.82 (0.71–0.95)
χ^2^ trend (*p*-value)					7.13 (0.008)
1-point increase					0.95 (0.92–0.99)

^a^ Adjusted for study centre, age, education, body mass index, physical activity, smoking, parity, menopausal status, oral contraceptive use, hormone-replacement therapy use, diabetes, family history of breast cancer, non-alcohol energy intake; ^b^ The sum does not add up to the total because of 3 missing values on score’s components. ^c^ Reference category.

**Table 3:** Odds ratios (ORs) and corresponding 95% confidence intervals (CIs) of breast cancer according to the Mediterranean diet score (MDS) across strata of selected covariates. Italy and Switzerland, 1991–2008.

	MDS			
	Cases/Controls, OR ^a^ (95% CI)			
	0–3	4–5	6–9	1-Point Increase ^b^
**Age group**				
<50	268/321	435/448	275/297	0.94 (0.88–0.99)
	1 ^c^	0.88 (0.70–1.11)	0.79 (0.61–1.03)	
50–59	268/276	398/427	283/283	0.97 (0.91–1.03)
	1 ^c^	0.87 (0.69–1.10)	0.87 (0.67–1.13)	
≥60	341/425	490/607	275/306	0.96 (0.91–1.02)
	1 ^c^	0.83 (0.68–1.02)	0.86 (0.67–1.10)	
	*p heterogeneity = 0.867*			*p heterogeneity = 0.911*
**Menopausal status**				
Pre-menopause	311/365	510/480	329/334	0.93 (0.88–0.99)
	1 ^c^	0.93 (0.75–1.16)	0.78 (0.61–1.00)	
Post-menopause	563/657	812/1002	504/552	0.97 (0.93–1.01)
	1 ^c^	0.83 (0.71–0.97)	0.87 (0.72–1.04)	
	*p heterogeneity = 0.150*			*p heterogeneity = 0.711*
**Education (years)**				
<7	299/380	586/734	387/467	0.98 (0.93–1.04)
	1 ^c^	0.93 (0.77–1.13)	0.94 (0.75–1.17)	
≥7	574/635	733/740	440/411	0.92 (0.88–0.96)
	1 ^c^	0.78 (0.66–0.93)	0.69 (0.56–0.85)	
	*p heterogeneity = 0.341*			*p heterogeneity = 0.181*
**Parity**				
0	183/230	191/249	130/118	0.93 (0.86–1.01)
	1 ^c^	0.70 (0.52–0.96)	0.74 (0.51–1.08)	
1–2	502/558	842/805	494/503	0.94 (0.90–0.98)
	1 ^c^	0.95 (0.80–1.12)	0.81 (0.67–0.99)	
≥3	191/233	288/428	209/264	0.97 (0.91–1.04)
	1 ^c^	0.74 (0.58–0.96)	0.82 (0.62–1.10)	
	*p heterogeneity = 0.149*			*p heterogeneity = 0.565*
**Body mass index (kg/m^2^)**				
<25.0	487/560	704/772	457/434	0.97 (0.92–1.01)
	1 ^c^	0.86 (0.72–1.03)	0.88 (0.72–1.08)	
≥25.0	390/462	619/710	376/452	0.93 (0.89–0.98)
	1	0.87 (0.72–1.05)	0.76 (0.61–0.94)	
	*p heterogeneity = 0.405*			*p heterogeneity = 0.820*
**Oral contraceptive use**				
Never	747/904	1121/1313	710/766	0.96 (0.92–0.99)
	1 ^c^	0.85 (0.74–0.97)	0.84 (0.72–0.98)	
Ever	130/118	202/169	123/120	0.91 (0.83–1.00)
	1 ^c^	0.87 (0.60–1.25)	0.65 (0.43–0.98)	
	*p heterogeneity = 0.187*			*p heterogeneity = 0.145*
**Hormone-replacement therapy use**				
Never	742/921	1198/1356	759/809	0.96 (0.93–1.00)
	1 ^c^	0.89 (0.78–1.02)	0.85 (0.73–0.99)	
Ever	135/101	125/126	74/77	0.87 (0.78–0.97)
	1 ^c^	0.68 (0.45–1.02)	0.62 (0.38–1.02)	
	*p heterogeneity = 0.018*			*p heterogeneity = 0.001*

^a^ Adjusted for study centre, age, education, body mass index, physical activity, smoking, parity, menopausal status, oral contraceptive use, hormone-replacement therapy use, diabetes, family history of breast cancer, non-alcohol energy intake, when appropriate; ^b^ OR for one point increase in the MDS; ^c^ Reference category.

## Supplementary Materials

The following are available online at http://www.mdpi.com/2072-6643/10/3/326/s1, Table S1: Distribution of 3034 cases of breast cancer and 3392 controls according to selected characteristics, in Italy and Switzerland, 1991–2008.
