# Supplementary material for: Mediterranean Diet and Breast Cancer Risk
Source: Nutrients. 2018 Mar 8;10(3):326. doi: 10.3390/nu10030326 (PMC5872744; doi:10.3390/nu10030326)
Supplement: Supplementary file 1 [file nutrients-10-00326-s001.docx]

**Supplementary Table 1.** Distribution of 3034 cases of breast cancer and 3392 controls according to selected characteristics, in Italy and Switzerland, 1991-2008.

|  | **Italy** | |  | **Switzerland** | | |
| --- | --- | --- | --- | --- | --- | --- |
| **Characteristic** | **Cases**  **n (%)** | **Controls**  **n (%)** |  | **Cases**  **n (%)** | **Controls**  **n (%)** |  |
| **Age group** |  |  |  |  |  |  |
| <40 | 206 (8.02) | 257 (9.93) |  | 54 (11.61) | 146 (18.16) |  |
| 40-44 | 264 (10.28) | 215 (8.31) |  | 38 (8.17) | 68 (8.46) |  |
| 45-49 | 369 (14.36) | 297 (11.48) |  | 47 (10.11) | 84 (10.45) |  |
| 50-54 | 403 (15.69) | 397 (15.34) |  | 79 (16.99) | 92 (11.44) |  |
| 55-59 | 406 (15.80) | 411 (15.88) |  | 61 (13.12) | 87 (10.82) |  |
| 60-64 | 393 (15.30) | 391 (15.11) |  | 52 (11.18) | 89 (11.07) |  |
| 65-69 | 340 (13.23) | 384 (14.84) |  | 73 (15.70) | 99 (12.31) |  |
| ≥70 | 188 (7.32) | 236 (9.12) |  | 61 (13.12) | 139 (17.29) |  |
| **Education (years)^a^** |  |  |  |  |  |  |
| <7 | 1259 (49.28) | 1569 (61.17) |  | 14 (3.01) | 14 (1.74) |  |
| 7-11 | 714 (27.95) | 642 (25.03) |  | 258 (55.48) | 478 (59.45) |  |
| ≥12 | 582 (22.78) | 354 (13.80) |  | 193 (41.51) | 312 (38.81) |  |
| **Menopausal status^a^** |  |  |  |  |  |  |
| Pre-/peri- menopause | 987 (38.55) | 842 (32.57) |  | 163 (35.13) | 338 (42.04) |  |
| Menopause at <50 years of age | 642 (25.08) | 845 (32.69) |  | 135 (29.09) | 173 (21.52) |  |
| Menopause at ≥50 years of age | 931 (36.37) | 898 (34.74) |  | 166 (35.78) | 293 (36.44) |  |
| **Parity^a^** |  |  |  |  |  |  |
| Nulliparae | 401 (15.63) | 380 (14.69) |  | 103 (22.15) | 217 (26.99) |  |
| 1 | 584 (22.76) | 494 (19.10) |  | 92 (19.78) | 194 (24.13) |  |
| 2 | 968 (37.72) | 909 (35.15) |  | 195 (41.94) | 270 (33.58) |  |
| 3 | 406 (15.82) | 489 (18.91) |  | 59 (12.69) | 94 (11.69) |  |
| ≥4 | 207 (8.07) | 314 (12.14) |  | 16 (3.44) | 29 (3.61) |  |
| **Family history of breast cancer in first-degree relatives** |  |  |  |  |  |  |
| No | 2310 (89.92) | 2467 (95.32) |  | 414 (89.03) | 782 (97.26) |  |
| Yes | 259 (10.08) | 121 (4.68) |  | 51 (10.97) | 22 (2.74) |  |

^a^ The sum does not add up to the total because of some missing values.
